# Chronic infusion of interleukin‐17 promotes hypertension, activation of cytolytic natural killer cells, and vascular dysfunction in pregnant rats

**DOI:** 10.14814/phy2.14038

**Published:** 2019-04-08

**Authors:** Olivia K. Travis, Dakota White, W. Austin Pierce, Ying Ge, Cassandra Y. Stubbs, Frank T. Spradley, Jan M. Williams, Denise C. Cornelius

**Affiliations:** ^1^ Department of Experimental Therapeutics and Pharmacologyogy University of Mississippi Medical Center Jackson Mississippi; ^2^ Department of Emergency Medicine University of Mississippi Medical Center Jackson Mississippi; ^3^ Department of Surgery University of Mississippi Medical Center Jackson Mississippi

**Keywords:** Hypertension, IL‐17, natural killer cells, pregnancy, vascular reactivity

## Abstract

Previous studies by our lab have established that placental‐ischemia stimulated T‐helper 17 cells (T_H_17s) cause increased cytolytic natural killer (cNK) cell proliferation and activation during pregnancy; however, the exact mechanism is unknown. The objective of this study was to investigate the role of interlukin 17 (IL‐17) in inducing cNK cell activation in pregnancy. We infused 150 pg/day of recombinant IL‐17 into a subset of normal pregnant (NP) Sprague Dawley rats from gestation day (GD) 12–19 (NP+IL‐17). On GD 19, mean arterial pressure (MAP), fetal and placental weights, cytokines, cNK cell activation, cytotoxic enzymes, and vascular reactivity were assessed. MAP significantly increased from 99 ± 3 mmHg in NP to 120 ± 1 mmHg in NP+IL‐17 (*P* < 0.05). Fetal weight significantly decreased from 2.52 ± 0.04 g in NP to 2.32 ± 0.03 g in NP+IL‐17 as did placental weight (NP: 0.65 ± 0.03 g; NP+IL‐17: 0.54 ± 0.01 g, *P* < 0.05). Plasma levels of TNF‐*α* increased to 281.4 ± 55.07 pg/mL in NP+IL‐17 from 145.3 ± 16.03 pg/mL in NP (*P* < 0.05) while placental levels of VEGF decreased from 74.2 ± 6.48 pg/mg in NP to 54.2 ± 3.19 pg/mg in NP+IL‐17. Total NK cells were increased in the placenta (NP: 14.3 ± 3.49%; NP+IL‐17: 29.33 ± 2.76%, *P* < 0.05) as were cytolytic NK cells (NP: 3.31 ± 1.25%; NP+IL‐17: 13.41 ± 1.81%, *P* < 0.05). A similar trend was observed in circulating NK cells. Plasma granzyme K increased from 3.55 ± 2.29 pg/mL in NP to 20.9 ± 7.76 pg/mL in NP+IL‐17 (*P* < 0.05), and plasma granzyme B increased from 10.95 ± 0.64 pg/mL in NP to 14.9 ± 0.98 pg/mL in NP+IL‐17(*P* < 0.05). In the placenta, both granzyme A (NP: 246.1 ± 16.7 pg/mg; NP+IL‐17: 324.3 ± 15.07 pg/mg, *P* < 0.05) and granzyme B (NP: 15.18 ± 3.79 pg/mg; NP+IL‐17: 27.25 ± 2.34 pg/mg, *P* < 0.05) increased in response to IL‐17 infusion. Finally, vascular reactivity of uterine arteries was significantly impaired in response to IL‐17 infusion. The results of this study suggest that IL‐17 plays a significant role in the activation of cNK cells during pregnancy.

## Introduction

Preeclampsia (PE) is a hypertensive disorder of pregnancy that affects 5–8% of pregnancies and is one of the leading causes of maternal and fetal morbidity worldwide (Roberts et al. 2012; Phipps et al. 2016). PE is characterized by new‐onset hypertension after the 20th week of pregnancy accompanied by indicators of end‐organ damage that can include proteinuria, thrombocytopenia, and pulmonary edema and usually results in impaired fetal growth due to intrauterine growth restriction (IUGR) (Backes et al. 2011; Roberts et al. 2012; Warrington et al. 2013). Despite decades of research, the exact cause of PE remains unclear; however, the leading theory suggests that insufficient invasion of the myometrium and endometrium by cytotrophoblasts is the initiating factor (Warrington et al. 2013; Phipps et al. 2016). The reduced invasion results in insufficient targeting and remodeling of maternal vessels including the spiral arteries, which is thought to drive placental ischemia/hypoxia‐induced release of antiangiogenic factors into the maternal circulation (Hladunewich et al. 2007; Young et al. 2010; Uzan et al. 2011). Some of these factors include soluble fms‐like tyrosine‐1 (sFLt‐1) and inflammatory cytokines such as IL‐1β, IL‐2, and IL‐18 (Raghupathy 2013). Collectively, these factors contribute to maternal endothelial dysfunction and the subsequent clinical manifestations of this hypertensive disorder (Wang et al. 2009; Laresgoiti‐Servitje 2013; Raghupathy 2013; Phipps et al. 2016).

It has been observed in clinical studies that the maternal immune system of preeclamptic women is altered with an excess of inflammatory molecules including TNF‐*α*, IL‐6, and IL‐17 and abnormal lymphocyte profiles (Toldi et al. 2011; Pinheiro et al. 2013; Udenze et al. 2015). Normal pregnancy is associated with a predominance of decidual natural killer (NK) cells with limited cytolytic activity and a high ratio of T regulatory (*T*_Reg_) to T helper 17 (T_H_17) cells in the circulation (Prins et al. 2009; Toldi et al. 2011; LaMarca et al. 2016; Taylor and Sasser 2017). In contrast to normal pregnancy, many studies have shown that NK cells in the decidua of preeclamptic women are composed of a higher cytolytic NK (cNK) cell population and a predominance of pro‐inflammatory T_H_17 cells along with a corresponding decrease in *T*_Reg_ number and function (Prins et al. 2009; Toldi et al. 2011; Darmochwal‐Kolarz et al. 2012; Laresgoiti‐Servitje 2013; Cornelius and LaMarca 2014).

The Reduced Uterine Perfusion Pressure (RUPP) rat model recapitulates many preeclamptic characteristics and has been used to better understand how aberrant immune activation contributes to the pathophysiology of PE (Li et al. 2012; Wallace et al. 2013; LaMarca et al. 2016). Our group has previously demonstrated that depletion of NK cells in the RUPP model improves both maternal and fetal outcomes (Elfarra et al. 2017). In addition, we have shown that adoptive transfer of RUPP stimulated T_H_17 cells into normal pregnant animals induces a preeclamptic phenotype and results in significantly increased cNK cell populations (Cornelius et al. 2016; Shields et al. 2018). However, the mechanism of how T_H_17 cells activate NK cells during PE has not been investigated. Al Omar et al. (2013) previously reported mRNA and protein expression of IL‐17 receptor C (IL‐17 RC) in human NK cells. Since T_H_17 cells, IL‐17, the main cytokine secreted by T_H_17 cells, and cNK cells are all elevated in PE (Korn et al. 2009; Molvarec et al. 2015; Tabarkiewicz et al. 2015; Abdel‐Moneim et al. 2018), we hypothesized that increased IL‐17 activates cNK cells during pregnancy as a mechanism of pathophysiology of PE. To test a role for IL‐17 to induce cNK cells, we chronically infused recombinant IL‐17 into pregnant rats and evaluated changes in placental NK cell activation and function, and placental reactive oxygen species (ROS). Furthermore, maternal circulating cytokines, maternal blood pressure, renal ROS, uterine artery endothelial function, and fetal outcomes were assessed.

## Materials and Methods

Timed pregnant Sprague‐Dawley rats purchased from Envigo RMS, Inc. (Indianapolis, IN) were used in this study. At Envigo, the animals were maintained on Teklad 2018S. In the Center for Comparative Research at the University of Mississippi Medical Center, the animals were housed in a temperature‐controlled room (23°C) with a 12:12‐h light‐dark cycle and maintained on Teklad 8640. All experimental procedures executed in this study were in accordance with the National Institute of Health guidelines for use and care of animals. All protocols were approved by the Institutional Animal Care and Use Committee at the University of Mississippi Medical Center.

### Infusion of IL‐17

All of our in vivo experiments were performed in rats weighing approximately 250–275 g. On day 12 of gestation (GD12), under isoflurane anesthesia, the mini osmotic pump implantation surgery was performed. Briefly, a midline incision was made and a mini osmotic pump (model 2002, Alzet Scientific Corporation, Cupertino, CA) infusing recombinant IL‐17A/F (150 pg/day, R&D Systems, Minneapolis, MN) was placed intraperitoneally for infusion from GD12‐19 (Dhillion et al. 2012; Cornelius et al. 2013).

### Measurement of mean arterial pressure in conscious rats

On GD 18, catheters (V3 tubing) were inserted into carotid arteries for the measurement of mean arterial pressure (MAP), tunneled to the back of the neck, and exteriorized under isoflurane anesthesia. On GD 19, rats were placed in individual restrainers. Conscious MAP was monitored with a pressure transducer and recorded continuously for 30 min after a 30‐min stabilization period. Subsequently, blood was collected under anesthesia, placental and fetal weights were recorded, and tissue samples were collected after sacrifice.

### Determination of circulating and placental NK cell populations using flow cytometry

Single‐cell suspensions of placental leukocytes were prepared, as previously described (Shields et al. 2018). Briefly, one placenta from each rat was homogenized and filtered through a 70‐*μ*m cell strainer and resuspended in 15 mL of Rosswell Park Memorial Institute medium (RPMI) (10% FBS). Whole blood was collected in an EDTA tube and diluted with 5 mL of RPMI. Peripheral blood mononuclear cells (PBMCs) and placental lymphocytes were isolated by centrifugation on a cushion of Ficoll‐Isopaque (Lymphoprep, Accurate Chemical & Scientific Corp., Westbury, NY) according to the instructions of the manufacturer. Single‐cell suspensions (1 × 10^6^ cells) were stained for flow cytometry after blocking with 10% goat and mouse serum. Antibodies used for flow cytometry were as follows: VioGreen anti‐CD3 (Miltenyi Biotec, Auburn, CA), anti‐ANK61 antibody (Abcam, ab36392), antimouse IgG FITC (Abcam, ab97239), anti‐ANK44 (Abcamab36388), and antimouse IgG AlexaFluor 405 (Abcam, ab175663). Flow cytometry was performed on the Miltenyi MACSQuant Analyzer 10 (Miltenyi) and analyzed using FlowLogic software (Innovai, Sydney, Australia). Lymphocytes were gated in the forward and side scatter plots. After doublet exclusion, additional gates were set using fluorescence minus one (FMO) controls. Results are expressed as % of cells in the gated lymphocyte population.

### Determination of placental and renal reactive oxygen species

Superoxide production in the placenta and renal cortex were measured using the lucigenin technique, as previously described by our lab (Shields et al. 2018). Briefly, rat placentas and 1 g of renal cortexes from NP and NP+IL‐17 rats were snap frozen in liquid nitrogen immediately after collection and stored at −80°C until further processing. Tissue samples were homogenized using the Bio‐Rad Cell Lysis Kit (Bio‐Rad, Hercules, CA) according to the manufacturer's instructions. The tissue lysate was incubated with lucigenin at a concentration of 5 *μ*mol/L. The samples were allowed to equilibrate for 15 min in the dark, and the luminescence was measured for 10 sec with a BioTek Plate Reader (BioTek, Winooski, VT). Luminescence was recorded as relative light units per minute (RLUs/min). An assay blank containing lucigenin with no homogenate was subtracted from the reading before transformation of the data. Each sample was read three times and the average was used for data transformation. The protein concentration was measured using a protein assay with BSA standards (Pierce, 169 Rockford, IL) and the data are expressed as RLU/min/mg protein.

### Measurement of circulating and placental cytokines and cytolytic proteins

Plasma and placental homogenates were analyzed for IL‐12, IL‐17, TNF‐*α*, IFN‐*γ*, MIP3‐*α*, and VEGF using the Bio‐Plex Pro Rat Cytokine 23 Plex Immunoassay Kit (Bio‐Rad, Hercules, CA) according to the manufacturer's instructions. Circulating and placental levels of the NK cell cytolytic proteins perforin, granzyme A, granzyme B, and granzyme K were measured using commercial ELISA kits (MyBioSource, San Diego, CA) according to the manufacturer's instructions. The protein concentration of the placentas was measured using a protein assay with BSA standards and the placental data are expressed as pg/mg.

### Isolation of natural killer cells

On the day of harvest, 2 placentas from each rat were collected and homogenized in 30 mL of RPMI (10% FBS) The mixture was passed through a 70 *μ*m filter and lymphocytes were isolated by centrifugation on a cushion of Ficoll‐Hypaque according to the instructions of the manufacturer. Anti‐CD3 and anti CD‐161 antibodies (BD Biosciences San Jose, CA) were biotinylated using the DSB‐X protein labeling kit (Life Technologies, Grand Island, NY), according to the manufacturer's instructions. The isolated lymphocytes were incubated with the biotin labeled anti‐CD3 and the CD3^+^ population was isolated using FlowComp Dynabeads Flexi Kit (Invitrogen, Oslo, Norway) according to the manufacturer's instructions. The supernatant containing the CD3^−^ population of lymphocytes was then incubated with biotin labeled anti‐CD161 antibody. This solution was incubated again with FlowComp Dynabeads and the CD3^−^ CD161^−^ supernatant was removed. The CD3^−^ CD161^+^ cells bound to the FlowComp Dynabeads were separated using release buffer and the biotin labeled antibodies were separated according to the manufacturer's protocol prior to culture and expansion. The collected cells were resuspended in NK cell activation media (RPMI, 10% FBS, 1% Pen/Strep, 2 ng/mL IL‐2), seeded at 3 × 10^5^ cells/well in a 6‐well plate, and allowed to expand for 48 h.

### Assessment of cytolytic activity

Cytolytic activity of isolated NK cells was assessed using the Cytotox 96 ^®^ Non‐Radioactive Cytotoxic Assay Kit (Promega, San Luis Obispo, CA) according to the manufacturer's instructions. YAC1 cells (ATCC^®^ Manassas, VA,) served as the target cells. The assay was performed using an effector to target ratio of 50:1 with a 5‐h incubation time (Lv et al. 2012). Cytotoxicity percentage was calculated using the following formula: (Experimental‐Effector Spontaneous‐Target Spontaneous)/(Target Maximum‐Target Spontaneous) × 100. The results are expressed as fold change in cytolytic activity.

### Measurement of uterine artery endothelial function

On GD 19, uterine vessels were collected under isoflurane anesthesia. The arteries were cleaned of perivascular fat, cut into concentric rings, and mounted for wire myography (620M, Danish Myo Technology). The vessel segments were constricted with 2 × 10^−6^ mol/L phenylephrine (Phe) and evaluated for endothelial‐dependent vasorelaxation using a 12‐point cumulative concentration response curve to acetylcholine (ACh: 1 × 10^−10^ mol/L to 3 × 10^−4^ mol/L). The results are expressed as % Phe constriction by using the following equation: [(maximum Phe response − ACh response)/maximum Phe response − baseline before Phe constriction)] × 100.

### Statistical analysis

All of the data are expressed as mean ± SEM. Statistical analyses of the vasorelaxation data were performed using two‐way ANOVA with repeated measures followed by a Sidak's multiple comparisons test. All remaining data were checked to be consistent with Gaussian distribution by D'Agostino‐Pearson normality test. Statistical analyses were performed using unpaired Student's *T*‐test or Mann–Whitney *U* test, where appropriate. A value of *P* < 0.05 was considered statistically significant.

## Results

### Total and cytolytic natural killer cell populations in plasma and placenta after IL‐17 infusion

Plasma IL‐17 increased from 8.89 ± 1.38 pg/mL in NP (*n* = 8) to 19.41 ± 4.14 pg/mL in NP+IL‐17 (*n* = 8, *P* < 0.05) confirming successful infusion. Placental IL‐17 was not changed between NP+IL‐17 and NP rats: NP: 11.09 ± 0.74 pg/mg (*n* = 7) versus NP+IL‐17: 8.99 ± 1.10 pg/mg (*n* = 8).

**Figure 1:**
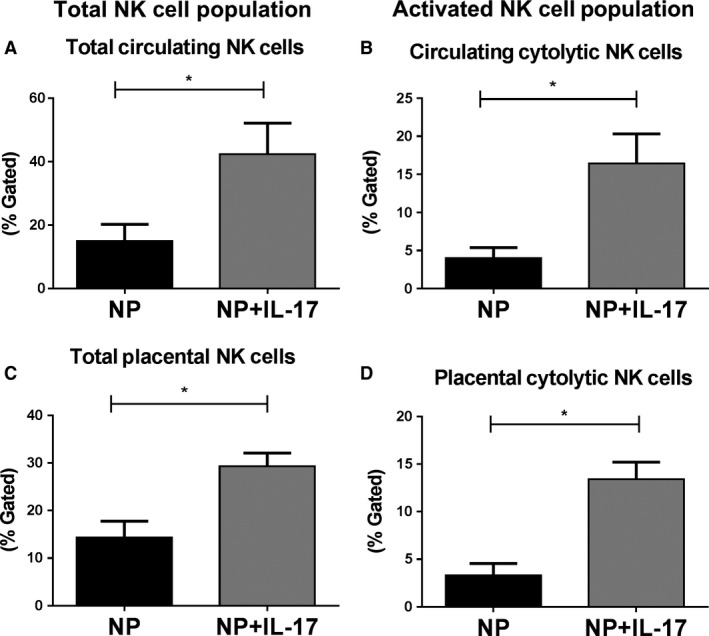
Effect of IL‐17 infusion on circulating and placental NK cell profiles in pregnant rats. Chronic IL‐17 infusion into normal pregnant (NP) rats results in increased total and cytolytic natural killer (NK) cells in the placenta and circulation. On GD 19, blood and placentas were collected and processed to obtain percentages of (A) circulating total NK cells and (B) circulating cytolytic NK cells along with (C) placental total NK cells, and (D) placental cytolytic NK cells through flow cytometry analysis. All data are expressed as mean ± SEM. Statistical analyses were performed using unpaired Student's *T*‐test. *P < 0.05 versus NP.

Flow Cytometry analysis of lymphocytes isolated from blood and placental samples was used to determine the % gated total and cytolytic NK cells in the circulation and placentas of animals from each group. Total Circulating NK cells increased significantly from 14.95 ± 5.30% in NP (*n* = 8) to 42.33 ± 9.82% in NP+IL‐17 (*n* = 8, *P* < 0.05, Fig. 1A). Similarly, the circulating cytolytic NK cell population significantly increased from 3.99 ± 1.40% in NP (*n* = 8) to 16.42 ± 3.91% in NP+IL‐17 (*n* = 8, *P* < 0.05, Fig. 1B). This trend was also reflected in the placenta with the total NK cell population significantly increasing from 14.3 ± 3.48% in NP (*n* = 8) to 29.33 ± 2.76% in NP+IL‐17 (*n* = 8, *P* < 0.05, Fig. 1C) and the cytolytic NK cell population significantly increasing from 3.31 ± 1.25% in NP (*n* = 8) to 13.41 ± 1.81% in NP+IL‐17 (*n* = 8, *P* < 0.05, Fig. 1D)

### Placental factors in response to IL‐17 infusion

**Figure 2:**
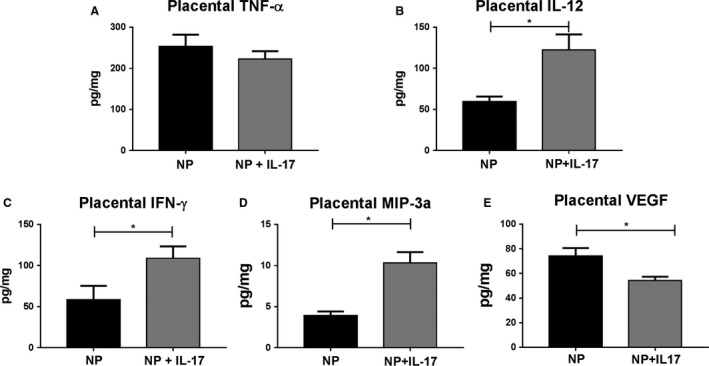
Effects of IL‐17 Infusion on placental cytokines in pregnant rats. Chronic IL‐17 infusion into normal pregnant (NP) rats results in increased inflammatory cytokines and decreased VEGF production in the placenta. On GD 19, placentas were collected and homogenized for analysis of placental cytokines. Placental levels of (A) TNF‐*α*, (B) IL‐12, (C) IFN‐*γ*, (D) MIP3*α*, and (E) VEGF normalized to total protein concentration are shown. All data are expressed as mean ± SEM. Statistical analyses were performed using unpaired Student's *T*‐test. *P < 0.05 versus NP

Placental levels of inflammatory factors TNF‐ *α*, IL‐12, IFN‐*γ*, MIP3‐*α*, and VEGF were measured in rats from each group. TNF‐*α* was unchanged between groups with 253.3 ± 28.78 pg/mg in NP (*n* = 7) versus 222.5 ± 19.33 pg/mg in NP+IL‐17 (*n* = 7, Fig. 2A). IL‐12 significantly increased to 122.6 ± 18.78 pg/mg in NP+IL‐17 (*n* = 8) from 29.54 ± 6.16 pg/mg in NP (*n* = 7, *P* < 0.05, Fig. 2B). IFN‐*γ* was significantly increased after IL‐17 infusion :(NP: 58.47 ± 16.82 pg/mg (*n* = 8), NP+IL‐17: 108.8 ± 14.45 pg/mg (*n* = 8), *P* < 0.05, Fig. 2C). Similarly, MIP3‐*α* increased from 3.92 ± 0.51 pg/mg in NP (*n* = 8) to 10.34 ± 1.30 pg/mg in NP+IL‐17 (*n* = 8, *P* < 0.05, Fig. 2D). Placental VEGF was significantly decreased from 74.18 ± 6.48 pg/mg in NP (*n* = 7) to 54.2 ± 3.19 pg/mg in NP+IL‐17 (*n* = 6, *P* < 0.05, Fig. 2E).

**Figure 3:**
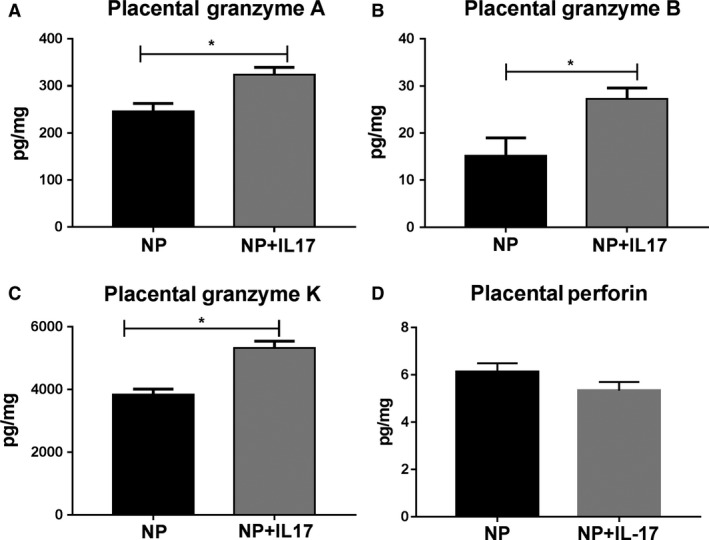
Effects of IL‐17 Infusion on placental cytolytic NK factors in pregnant rats: Cytolytic natural killer (NK) cell enzymes are increased in the placenta in response to chronic IL‐17 infusion in normal pregnant (NP) rats. Granzymes and perforin were measured in the placentas of animals from both groups. Placental levels of (A) granzyme A, (B) granzyme B, (C) granzyme K, and (D) perforin normalized to total protein concentration are shown. All data are expressed as mean ± SEM. Statistical analyses were performed using unpaired Student's *T*‐test or Mann–Whitney *U* test, where appropriate. *P < 0.05 versus NP.

Cytolytic enzymes were measured in placentas of animals from both groups to evaluate cNK cell activity. Granzyme A was significantly increased from 246.1 ± 16.7 pg/mg in NP (*n* = 7) to 324.3 ± 15.07 pg/mg in NP+IL‐17 (*n* = 7, *P* < 0.05 Fig. 3A). Similarly, granzyme B increased from 15.18 ± 3.79 pg/mg in NP (*n* = 8) to 27.25 ± 2.34 pg/mg in NP+IL‐17 (*n* = 7, *P* < 0.05, Fig. 3B). Placental levels of granzyme K significantly increased from 3830 ± 181.8 pg/mg in NP (*n* = 8) to 5324 ± 214.3 NP+IL‐17 (*n* = 8, *P* < 0.05, Fig. 3C). Placental levels of perforin were unchanged between groups; NP: 6.12 ± 0.37 pg/mg (*n* = 8), NP+IL‐17: 5.34 ± 0.36 pg/mg (*n* = 8, Fig. 3D)

**Figure 4:**
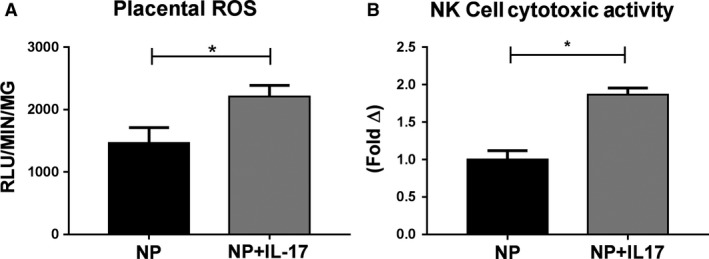
Effects of IL‐17 Infusion on Placental Oxidative Stress and NK cytotoxicity in pregnant rats. Chronic IL‐17 infusion into normal pregnant (NP) rats causes increased oxidative stress in the placenta and results in increased cytolytic activity of placental natural killer (NK) cells. (A) Superoxide production in the placenta was analyzed using the lucigenin assay. The results are expressed as Relative Light Units (RLUs)/min/mg. (B) The cytolytic activity of isolated placental NK cells was measured using a cytotoxicity assay based on lactate dehydrogenase (LDH) release. The results are expressed as fold change in cytolytic activity. All data are expressed as mean ± SEM. Statistical analyses were performed using unpaired Student's *T*‐test or Mann–Whitney *U* test, where appropriate. *P < 0.05 versus NP.

Placental ROS values nearly doubled from 1466  ± 244.5 RLU/min/mg in NP (*n* = 7) to 2210 ± 179.9 RLU/min/mg in NP+IL‐17 (*n* = 7, *P* < 0.05, Fig. 4A).

### Cytolytic function of placental NK cells in response to chronic IL‐17

The cytolytic function of isolated placental NK cells significantly increased in response to IL‐17 infusion. The in vitro cytolytic activity of placental NK cells increased by 90% in NP+IL‐17 (*n* = 4) compared to NP controls (*n* = 4, *P* < 0.05, Fig. 4B).

### Circulating factors in response to chronic IL‐17

Immune factors TNF‐*α*, IL‐12, IFN‐*γ*, MIP3‐*α*, and VEGF were measured in the plasma of animals from both groups (Table 1). Circulating levels of TNF‐*α* were significantly increased to 281.4 ± 55.07 pg/mL in NP+IL‐17 (*n* = 6) compared to 145.3 ± 16.03 pg/mL in the NP (*n* = 6, *P* < 0.05). Plasma IL‐12 values were unchanged between groups with 72.12 ± 13.25 pg/mL in NP (*n* = 7) compared to 117.9 ± 35.22 in NP+IL‐17 (*n* = 7). IFN‐*γ* showed a trending increase in response to IL‐17. Plasma IFN‐*γ* was 23.22 ± 5.55 pg/mL in NP rats (*n* = 8) versus 49.82 ± 13.11 pg/mL in NP+IL‐17 rats (*n* = 8, *P* = 0.08). There were no significant changes in plasma MIP3‐*α* between groups: NP: 4.16 ± 0.60 pg/mL (*n* = 7) versus NP+IL‐17: 7.66 ± 1.98 pg/mL (*n* = 7). Similarly, plasma VEGF was not significantly altered IL‐17 infusion: VEGF: NP: 18.36 ±  3.83 pg/mL (*n* = 7) versus NP+IL‐17: 42.12 ± 12.32 pg/mL (*n* = 7).

**Table 1:** Effects of IL‐17 Infusion on circulating cytokines and cytolytic NK factors in pregnant rats

Circulating cytokines and cytolytic proteins			
Cytokine/protein (pg/mL)	NP	NP+IL‐17	*P* value
TNF‐*α*	145.3 ± 16.03	281.4 ± 55.07^a^	*P* > 0.05
IL‐12	72.12 ± 13.25	117.9 ± 35.22	*P* = 0.15
IFN‐*γ*	23.22 ± 5.55	49.82 ± 13.11	*P* = 0.08
MIP‐3*α*	4.16 ± 0.60	7.66 ± 1.98	*P* = 0.12
VEGF	18.36 ± 3.83	42.12 ± 12.32	*P* = 0.07
Granzyme A	647.6 ± 147	901.4 ± 244.5	*P* = 0.61
Granzyme B	10.95 ± 0.64	14.9 ± 0.98^a^	*P* < 0.05
Granzyme K	3.55 ± 2.29	20.9 ± 7.76^a^	*P* < 0.05
Perforin	179.7 ± 22.48	176.2 ± 10.46	*P* = 0.88

Levels of cytokines were measured from the blood of each animal using the Bio‐Plex Pro Rat Cytokine 23 Plex Immunoassay Kit. Cytolytic granzymes and perforin were measured in the blood of each animal using commercial ELISAs. All data are expressed as mean ± SEM. Statistical analyses were performed using unpaired Student's *T*‐test.

a *P* < 0.05 versus NP.

Circulating levels of cytolytic NK enzymes were also measured in each group. There was no change in plasma levels of granzyme A with NP: 647.6 ± 147 pg/mL (*n* = 7) versus NP+IL‐17: 901.4 ± 244.5 pg/mL (*n* = 7). Importantly, circulating levels of granzyme B were significantly increased from 10.95 ± 0.64 pg/mL in NP (*n* = 6) to 14.9 ± 0.98 pg/mL in NP+IL‐17 (*n* = 6, *P* < 0.05) and levels of granzyme K significantly increased from 3.55 ± 2.29 pg/mL in NP (*n* = 7) to 20.9 ± 7.76 pg/mL in NP+IL‐17 (*n* = 8, *P* < 0.05). Plasma perforin was unchanged between groups; NP: 179.7 ± 22.48 pg/mL(*n* = 7), NP+IL‐17: 176.2 ± 10.46 pg/mL (*n* = 8).

### Mean arterial pressure, renal oxidative stress, and uterine artery function after chronic IL‐17 infusion

**Figure 5:**
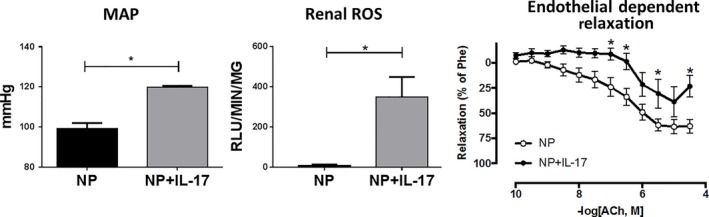
Effects of IL‐17 Infusion on MAP, Renal Oxidative Stress, and Uterine Artery Function. Chronic Infusion of IL‐17 results in increased mean arterial pressure (MAP), increased renal oxidative stress, and decreased uterine artery vasorelaxation. (A) On GD 19, Conscious mean arterial pressure (MAP) was measured via carotid catheters. Statistical analyses were performed using unpaired Student's *T*‐test. *P < 0.05 versus NP (B) Renal cortex tissue was collected and homogenized for analysis of oxidative stress. Superoxide production from the kidney was analyzed using the lucigenin assay. The results are expressed as Relative Light Units (RLUs)/min/mg. Statistical analyses were performed using Mann–Whitney *U* test. *P < 0.05 versus NP (C) Isolated uterine arteries were constricted with 2 × 10^−6^ mol/L phenylephrine (Phe) and subjected to a 12‐point cumulative concentration response curve to acetylcholine (ACh: 1 × 10^−10^ mol/L to 3 × 10^−4^ mol/L). The results are expressed as %Phe constriction using the following formula: [(maximum Phe response − ACh response)/(maximum Phe response − baseline before Phe constriction)] × 100. Statistical analyses were performed using two‐way ANOVA with repeated measures followed by Sidak's multiple comparisons test. *P < 0.05 versus NP.

Mean arterial pressure increased significantly from 99 ± 3 mmHg in NP (*n* = 8) to 120 ± 1 mmHg in NP+IL‐17 (*n* = 8, *P* < 0.05, Fig. 5A). Renal ROS values also increased from 7.94 ± 6.05 RLU/min/mg in NP (*n* = 6) to 350.4 ± 99.01 RLU/min/mg in NP+IL‐17 (*n* = 6), a change that was nearly 50‐fold (*P* < 0.05, Fig. 5B).

Cumulative concentration response curves to ACh were generated to assess endothelial dependent vasorelaxation in NP and NP+IL‐17 animals. Isolated uterine arteries of NP+IL‐17 demonstrated a significantly impaired vasorelaxation response in the presence of Ach compared to NP as determined by repeated measurements (*P* < 0.05). Additionally, the maximum relaxation response to Ach (3 × 10^−4^ mol/L) was significantly decreased from 63 ± 7% in NP (*n* = 13) to 23 ± 11% in NP+IL‐17 (*n* = 7, *P* < 0.05, Fig. 5C).

### Fetal and placental weight after chronic IL‐17 infusion during pregnancy

Reduced fetal weights were observed in NP+IL‐17. Average fetal weight significantly decreased from 2.52 ± 0.04 g in NP (*n* = 8) to 2.32 ± 0.03 g in NP+IL‐17(*n* = 8, *P* < 0.05). Similarly, placental weight was significantly decreased in NP+IL‐17 (0.54 ± 0.01 g, *n* = 8) compared to NP (0.65 ± 0.03 g, *n* = 8, *P* < 0.05).

## Discussion

We hypothesized that IL‐17 activates cNK cells during pregnancy as a mechanism of pathophysiology of PE. Our data demonstrate a significant increase in total and cytolytic NK cells in the circulation and placentas of pregnant rats infused with recombinant IL‐17 protein. Furthermore, we observed significant increases in placental inflammatory cytokines, cytolytic proteins, and ROS in response to chronic IL‐17. Finally, significant increases in blood pressure and renal ROS, and impaired vascular endothelial function were observed in pregnant rats after infusion of IL‐17. Cytolytic activation of NK cells by IL‐17 may be an important contributor of pathophysiology in PE.

NK cells are a class of lymphocytes known for their ability to recognize and kill cells without the need for prior sensitization typically via release of granzymes or perforin (Fukui et al. 2011). Decidual NK cells have been recognized as a distinct subtype with these cells comprising nearly 40 % of decidual cells in early pregnancy before decreasing to low levels by the last trimester (Sargent et al. 2007; Jabrane‐Ferrat and Siewiera 2014). They are distinct in that they show little cytolytic activity, are major secretors of angiogenic and trophoblast attracting cytokines such as VEGF, IL‐8, and IL‐10, and play an important role in promoting tolerance to cytotrophoblasts and directing their invasion of and subsequent remodeling of maternal vessels in the myometrium and endometrium (Hanna et al. 2006; Saito et al. 2007; Fu et al. 2013; Jabrane‐Ferrat and Siewiera 2014). Several studies have shown that dNK cells of women with PE are significantly increased during late pregnancy and even at parturition (Stallmach et al. 1999; Wilczynski et al. 2003; Bachmayer et al. 2006). They also become activated and display an increased cytolytic type 1 phenotype compared to NK cells of normal pregnant women; however, the exact mechanism of this activation is unknown (Borzychowski et al. 2005; Sargent et al. 2007; Fukui et al. 2011). In the current study, we attempt to elucidate what is inducing this change in NK cells.

In our previous studies, we found that the preeclamptic RUPP rat model had significant increases in cNK cells in the circulation and placenta. Additionally, NK depletion improved maternal and fetal outcomes indicating that this activation contributed to the preeclamptic phenotype (Elfarra et al. 2017). We next performed an adoptive transfer of RUPP stimulated T_H_17 cells into normal pregnant animals and saw increased NK cell proliferation and production of cytolytic enzymes. We hypothesized that this was a result of increased ROS production, but treatment with TEMPOL had no effect on the NK cell activity (Shields et al. 2018). Since NK cells express the IL‐17 RC receptor and IL‐17, the major cytokine produced by T_H_17 cells, is reported to be increased in PE women (Molvarec et al. 2015), we hypothesized that IL‐17 stimulates increased NK cell proliferation and cytolytic activation. Much of the research examining the relationship between NK cells and IL‐17 has been done outside the perspective of pregnancy. Several studies have identified IL‐17 induction of NK cell cytolytic activity in the context of cancer and systemic fungal infections (Kryczek et al. 2009; Bär et al. 2014; O'Sullivan et al. 2014). In terms of pregnancy, an in vitro study on human NK cells by Al Omar et al. (2013) found that IL‐17 caused increased cytolytic activity, but there have been no in vivo studies further examining this effect. We decided to test our hypothesis by infusing IL‐17 into pregnant animals and found significant increases in both circulating and placental NK cell number and production of cytolytic enzymes. Within the placenta, we also see a significant increase in NK cytolytic activity and inflammatory cytokines. These data suggest that T_H_17 cells may induce NK activation through IL‐17 secretion in PE.

In this study, we thoroughly evaluate cNK activation using several methods. Through flow cytometry analysis, we see a significant increase in cNKs in both the placenta and circulation. (Fig. 1B and D). Importantly, we analyzed in vitro cytolytic activity of isolated placental NK cells using a cytotoxicity assay based on lactate dehydrogenase (LDH) release, and we observe an increase in cytolytic activity of isolated placental NK cells from the IL‐17 infusion group (Fig. 4B). We also see increased levels of NK cytolytic granzymes A, B, and K that indicate increased cNK activity (Fig. 3) and may be responsible for the observed increase in placental ROS. IL‐17 has been shown to directly recruit neutrophils and increase their activity resulting in increased oxidative stress (Amulic et al. 2012; Bär et al. 2014). Additionally, several studies have found that cytolytic granzymes A, B, and K also result in significant ROS production through their degradation of mitochondrial proteins (Martinvalet et al. 2005; Guo et al. 2008; Jacquemin et al. 2015; Martinvalet 2015). Therefore, the results of our experiments provide evidence that IL‐17 may also cause increased ROS through cNK activation.

The enhanced inflammatory cytokine profile in the placenta also supports a shift of NK cells toward a type1 phenotype. In this study, we see increased IL‐12 within the placenta of the IL‐17 infusion group (Fig. 2B), and IL‐17 has been observed to increase in vitro production of IL‐12 by peripheral blood mononuclear cells (PBMC's) (Wu et al. 2015). IL‐12 activates NK cells and stimulates them to produce IFN‐*γ* (Eickhoff et al. 2013; Lusty et al. 2017) that is also increased in the placentas of our IL‐17 infusion group (Fig. 2C). Importantly, the increased IL‐12 could also stimulate T_H_1 cells to produce IFN‐*γ* as well. However, T_H_1s were not measured in this study. MIP‐3*α* is a chemoattractant that induces NK chemotaxis to sites of inflammation. This chemokine is expressed by T_H_17 cells, regulated by IL‐17 (Gaffen 2008), and is increased in the placentas of our NP+IL‐17 animals (Fig. 2D). The presence of MIP‐3 alpha may explain the enhanced presence of NK cells in the placentas of the NP+IL‐17 animals on GD 19 although IL‐17 is unchanged in the placenta.

The significant decrease in placental VEGF observed in the IL‐17 infusion group of rats also suggests a phenotypic change in the NK cells (Fig. 2E). During normal pregnancy, dNK cells are major producers of VEGF (Jabrane‐Ferrat and Siewiera 2014; Kwak‐Kim et al. 2014. However, cNK cells produce less VEGF (Zhang et al. 2013) and this may contribute to the decreased VEGF seen in the placental homogenates of the IL‐17 infusion rats. Studies have documented decreased production of VEGF in placentas (Zhou et al. 2010) and isolated PBMC's (Cardenas‐Mondragon et al. 2014) of preeclamptic women compared to normal pregnant women. It has also been well established that VEGF is important for proper endothelial function (Kroll and Waltenberger 2000; Kliche and Waltenberger 2001). An in vitro study by Zhou et al. (2010) revealed that HUVEC cells demonstrated decreased proliferation and nitric oxide synthesis when VEGF levels were decreased via siRNA transfection. Therefore, decreased production of VEGF may play a role in the impaired endothelial dependent vasorelaxation of isolated uterine arteries from the IL‐17 infusion group (Fig. 5C).

This study shows that chronic infusion of IL‐17 induces circulating and placental NK cell activation and proliferation. However, some limitations to the study warrant further investigation. While we see cNK activation in both the placenta and circulation, we did not observe a significant change in placental levels of IL‐17. This may be due to the cytokine being internalized and metabolized by targeted cells in the placenta after signal transduction. Additionally, it could be that the IL‐17 is only increased in the plasma in this model, and activated NK cells are being recruited to the placenta by the increased placental levels of MIP3*α*. Finally, due to the observed increase in renal ROS production (Fig. 5B) and the established role of the kidney in chronic blood pressure regulation, an investigation of renal NK cell profiles may provide more insight into IL‐17's effects.

This study establishes an important role of IL‐17 in NK cell proliferation and activation in pregnancy. While several studies have identified T_H_17 cells and IL‐17 as contributors to the pathophysiology of preeclampsia (Dhillion et al. 2012; Cornelius and LaMarca 2014) the mechanism behind this has not been fully determined. From our previous studies, we have identified that the effects are due in part to increased ROS production (Cornelius and LaMarca 2014), but here we show that direct stimulation of NK proliferation and activation present another arm of the role of IL‐17 in preeclampsia. It also suggests that targeting IL‐17 secretion may be a viable therapeutic option in preventing excessive cytolytic NK activity within the placenta.

## Conflict of Interest

No conflicts of interest, financial or otherwise, are declared by the authors.
